# Comprehensive Analyses of MELK-Associated ceRNA Networks Reveal a Potential Biomarker for Predicting Poor Prognosis and Immunotherapy Efficacy in Hepatocellular Carcinoma

**DOI:** 10.3389/fcell.2022.824938

**Published:** 2022-05-27

**Authors:** Yu Liu, Rongkuan Li, Xiaobo Wang, Zuguang Xue, Xiaozhou Yang, Bo Tang

**Affiliations:** ^1^ Department of Infectious Disease, The Second Affiliated Hospital of Dalian Medical University, Dalian, China; ^2^ Department of Hematology, The Second Affiliated Hospital of Dalian Medical University, Dalian, China

**Keywords:** MELK, ceRNA network, immune cell infiltration, biomarker, immunotherapy, hepatocellular carcinoma

## Abstract

**Background:** Hepatocellular carcinoma (HCC) is one of the most common malignant tumors in the world with high morbidity and mortality. Identifying specific molecular markers that can predict HCC prognosis is extremely important. MELK has been reported to play key roles in several types of human cancers and predict poor prognosis. This study was aimed to explore the impact of MELK on HCC.

**Methods:** A pan-cancer analysis of MELK was conducted by The Cancer Genome Atlas (TCGA) and the Genotype-Tissue Expression (GTEx) data. The prognosis of MELK in various cancers was analyzed in GEPIA. Then, a ceRNA network of MELK was constructed based on the comprehensive consideration of the expression analysis, the correlation analysis, and the survival analysis by R software. The correlation of MELK and immune cell infiltration was analyzed by TIMER and ﻿CIBERSORT. Then, the overall survival of differentially expressed immune cells was conducted. The correlation of MELK and immune checkpoints expression was analyzed by GEPIA.

**Results:** MELK was overexpressed in 14 types of human cancers, and its expression was significantly higher than that in both unmatched and paired normal samples in HCC. Higher MELK expression was correlated with poorer survival and advanced clinical stage, topography (T) stage, and histological grade. The univariate and multivariate Cox regression analyses showed that MELK was an independent risk factor for poor prognosis in HCC. Then, we constructed a ceRNA network consisting of MELK, miR-101-3p, and two lncRNAs (SNHG1 and SNHG6) after evaluating the expression and impact on prognosis in HCC of these RNAs. TIMER and CIBERSORT databases indicated that MELK was correlated with various immune cells including B cells, CD8^+^ T cells, CD4^+^ T cells, macrophage, neutrophil, and dendritic cells in HCC. Of them, B cells, CD4^+^ T cells, macrophage, and neutrophil were related to the prognosis of HCC. In addition, MELK was significantly positively correlated with the immune checkpoint genes.

**Conclusions**: MELK may be a novel potential biomarker for predicting prognosis and immunotherapy efficacy in patients with HCC. Our study may provide new molecular and therapeutic strategies for the treatment of HCC patients.

## Introduction

Primary liver cancer is the sixth most commonly diagnosed cancer and the third leading cause of cancer death worldwide in 2020, with approximately 906,000 new cases and 830,000 deaths (Sung et al., 2021). HCC is the main type of primary liver cancer, comprising 75–85% of cases (Llovet et al., 2012). In contrast to the decreasing disease burden and impact of many other major cancers, the overall burden of HCC is increasing worldwide over time (Yang et al., 2019). Due to lack of specific clinical manifestations in the early stage, many HCC patients are diagnosed at advanced stages when they suffer from an aggravated condition and miss the best timing of treatment. Although the diagnostic and therapeutic approaches of HCC have gradually improved, the overall survival of the patients with HCC remains very low. The recurrence is still common and elusive, even for the patients who received successful surgical excision or liver transplantation. Given the lack of early diagnostic methods, it is often difficult to detect the early recurrence and metastasis. Immune checkpoint inhibitors (ICIs) have already shown systemic and durable anti-tumor responses in many types of cancers including HCC (Federico et al., 2020). However, the immune-related adverse events during the ICI therapy can affect any organ system and in some cases can be lethal (Postow et al., 2018). Therefore, early prediction for the HCC progression as well as the immunotherapy efficacy is of great importance to improve the prognosis of HCC patients.

Maternal embryonic leucine zipper kinase (MELK) was a member of the snfl/AMPK kinase family, encoding a serine/threonine kinase that is highly conserved across a variety of mammalian and non-mammalian species. Unlike most members of this family, only functioning in cell survival under metabolically challenging conditions, MELK participates in diverse processes, including cell cycle, cell proliferation, apoptosis, RNA processing, and embryonic development (Davezac et al., 2002; Vulsteke et al., 2004; Jung et al., 2008). Furthermore, MELK is involved in multiple protein interactions that affect many stages of tumorigenesis (Chung et al., 2012). Recent studies indicated that MELK was highly expressed in several human cancers, including breast cancer, prostate cancer, gastric cancer, liver cancer, glioblastoma multiforme, and lung cancer (Gray et al., 2005; Xia et al., 2015). Furthermore, overexpression of MELK has been markedly associated with decreased survival and poor prognosis for some tumors (Gray et al., 2005; Sun et al., 2021). However, a comprehensive study to identify the role and mechanism of MELK in HCC is still absent. Also, the impact of MELK on the tumor immune infiltration in HCC is still not evaluated.

The competitive endogenous RNA (ceRNA) hypothesis was first proposed in 2011 and suggested that the long non-coding RNAs (lncRNAs) could act as ceRNAs to bind to microRNAs (miRNAs) and affect the regulation of miRNAs on target mRNAs, thus regulating the expression of related target genes (Cao et al., 2018). In this study, we performed expression analysis, correlation analysis, and survival analysis for MELK in HCC using TCGA database. Then, a ceRNA network consisting of MELK, lncRNAs, and miRNAs was established. Finally, we explored the association of MELK with the immune cell infiltration, markers of immune cells, and immune checkpoints expression in HCC. In summary, our work suggested that ncRNA-mediated overexpression of ﻿MELK was correlated with poor prognosis, tumor immune infiltration, and immunotherapy efficacy of patients in HCC. Our study may provide new molecular and therapeutic strategies for the treatment and prognosis of HCC patients.

## Materials and Methods

### The Cancer Genome Atlas Data Download and Analysis

The RNA-seq data and clinical data of 33 types of cancer were downloaded from TCGA database (https://portal.gdc.cancer.gov/). The RNA expression data from different patients were integrated separately into files. Duplicated samples were removed from the RNA expression matrix, and the expression data of duplicated genes were averaged. The expression analysis of MELK was performed in pan-cancer using the R package *limma*, and *p* < 0.05 was considered statistically significant. Then, the RNA expression data and the clinical characteristics of 421 samples, including 371 tumor samples and 50 adjacent non-tumor samples, were obtained from TCGA-LIHC dataset.

### GEPIA Database Analysis

GEPIA (http://gepia.cancer-pku.cn/) is a public data platform containing gene expression data and patient characteristics for the majority of human tumors based on TCGA and GTEx data (Tang et al., 2017). We used this web tool to perform the comparison of the MELK expression between tumor and normal groups. *p* value < 0.05 and log_2_FC > 1 were considered statistically significant. GEPIA was also employed to conduct the overall survival (OS) and disease-free survival (DFS) analysis for MELK in various human cancers. In addition, the correlation of MELK and markers of various immune cells in HCC was also evaluated using the GEPIA database. |R| >0.1 and *p* value < 0.05 were considered statistically significant.

### Survival Analysis

The OS time and OS status in the clinical phenotype data from TCGA were used to perform survival analysis. The Cox proportional hazard model was performed to estimate whether the expression of MELK was related to patient prognosis. Hazard ratios (HR) > 1 and *p* < 0.05 indicated a significant relationship between MELK and increased risk of death. Kaplan–Meier (KM) analysis based on the log-rank test was conducted using R software (version 4.1.1). The plotted curves were visualized using a ggsurvplot R package. A *p*-value < 0.05 was considered to represent a significant difference in survival.

### Constructing the ceRNA Network

A co-expressed regulatory network comprised MELK, miRNA, and lncRNA was constructed to explore the potential functions of MELK in HCC. Based on the hypothesis, the candidate lncRNA and mRNA expression must have the same expression variation trend in HCC, while miRNA and mRNA expression should have the opposite trend. We predicted the miRNA–lncRNA interactions and MELK–miRNA interactions based on starBase 3.0 (http://starbase.sysu.edu.cn/). The differentially expressed miRNAs and lncRNAs between HCC samples and normal liver samples in TCGA-LIHC were identified using the *limma* package in R with a |log_2_FC | > 1 and adjusted *p*-value < 0.05 threshold. To improve the reliability, the Spearman’s correlation analysis was conducted between the expression of MELK–miRNA and miRNA–lncRNA individually. The criteria were set at r < −0.2 and FDR < 0.05. Finally, we established a ceRNA network by matching lncRNA–miRNA and miRNA–MELK pairs.

Then, we conducted OS analysis to assess the prognostic value of miRNA and lncRNA in the constructed ceRNA network. The surv_cutpoint function of the *survminer* package in R was used to determine the optimal cutoff value of the candidate miRNA and lncRNA. The KM survival curve was plotted to analyze the difference in survival between patients in high- and low-expression groups.

### Evaluation of Tumor Immune Cell Infiltration

The TIMER database (https://cistrome.shinyapps.io/timer/) is a web database for comprehensive analysis of tumor-infiltrating immune cells (Uhlen et al., 2017). Correlations between the MELK expression and various immune cell types, including B cells, neutrophils, CD4^+^ T cells, macrophages, CD8^+^ T cells, and dendritic cells, were estimated using TIMER. Then, we analyzed the correlation of MELK expression and the immune checkpoints expression in HCC. In addition, we estimated the proportion of 22 kinds of immune cell types in HCC patients by CIBERSORT. The *p* value and root mean-squared error were calculated for each sample. Samples with *p* < 0.05 were selected for further analysis. The immune cell abundance in different MELK expression groups was compared using the Wilcoxon rank-sum test. A KM survival curve analysis was performed to evaluate the OS of different immune cells in HCC patients.

### Statistical Analysis

Data were processed and analyzed by Perl and R software. The MELK expression in unmatched HCC samples and normal samples and matched HCC samples and adjacent normal samples were analyzed by Wilcoxon rank-sum tests and Wilcoxon signed-rank tests, individually. Comparisons of clinicopathological characteristics were performed *via* Wilcoxon rank-sum tests for quantitative variables and chi-square or Fisher’s exact tests for categorical variables. Differences among multiple groups were analyzed by the Kruskal–Wallis test. The correlation analysis was conducted by the Spearman’s correlation. Univariate and multivariate Cox regression analyses were performed to find independent variables related to prognosis.

## Results

### Pan-Cancer Analysis of MELK Expression in The Cancer Genome Atlas Database

**FIGURE 1:**
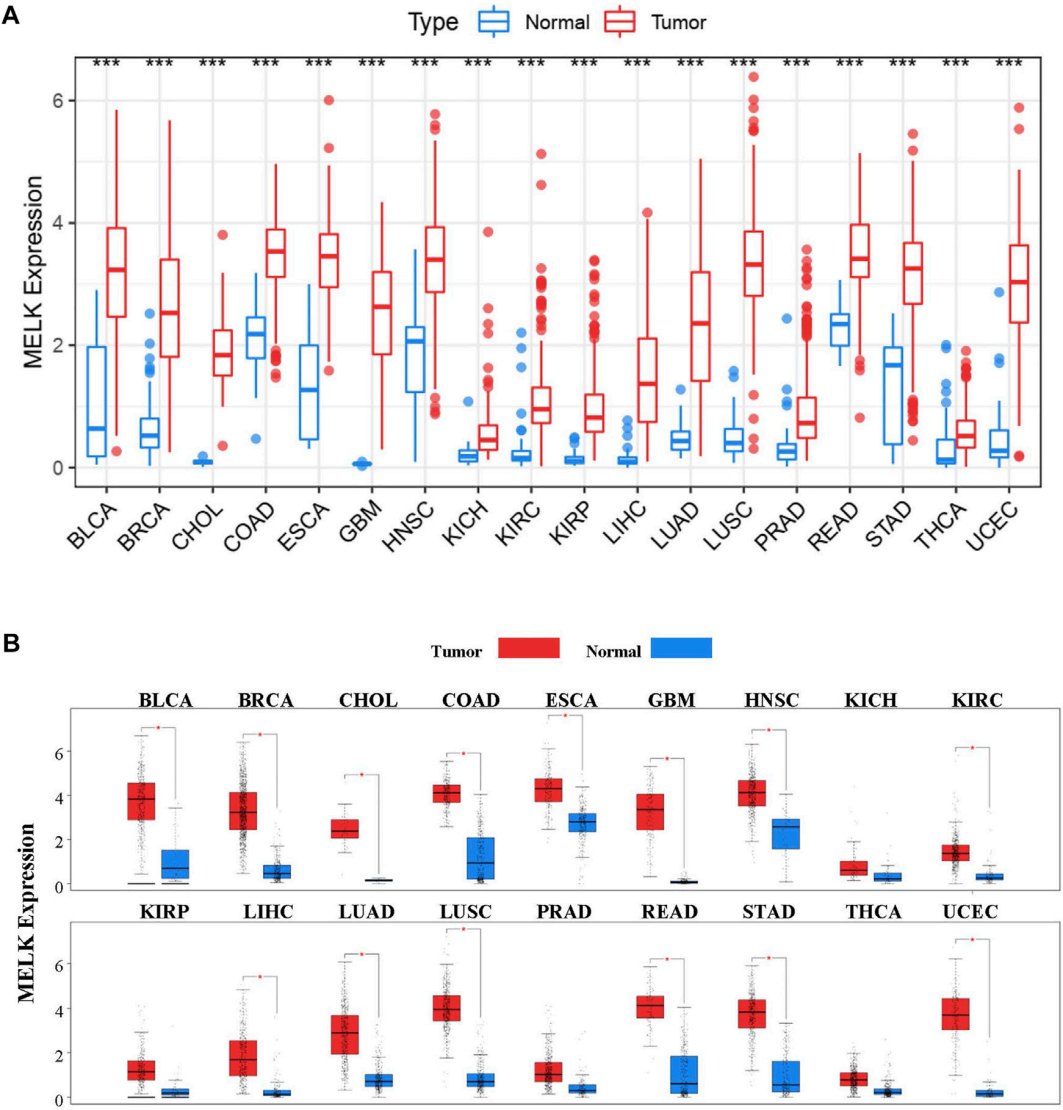
Pan-cancer analysis of MELK expression in TCGA database. **(A)** Expression of MELK in diverse cancers compared with the corresponding normal samples in TCGA database. **(B)** Expression of MELK in 18 types of human cancer based on TCGA and GTEx normal tissues. *p value < 0.05; **p value < 0.01; ***p value < 0.001.

To explore the possible roles of MELK in carcinogenesis, we compared its expression between tumor and normal tissues in 33 types of human cancer in TCGA database. As shown in Figure 1A, the MELK has a significant higher expression in 18 human cancers including BLCA, BRCA, CHOL, COAD, ESCA, GBM, HNSC, KICH, KIRC, KIRP, LIHC, LUAD, LUSC, PRAD, READ, STAD, THCA, and UCEC. To further validate the expression of MELK in 18 types of human cancer, the GEPIA database consisting of TCGA data and the GTEx data was employed. As shown in Figure 1B, the MELK expression was significantly increased in BLCA, BRCA, CHOL, COAD, ESCA, GBM, HNSC, KIRC, LIHC, LUAD, LUSC, READ, STAD, and UCEC compared with the normal group.

### The Impact on Prognosis of MELK in Pan-Cancer

**FIGURE 2:**
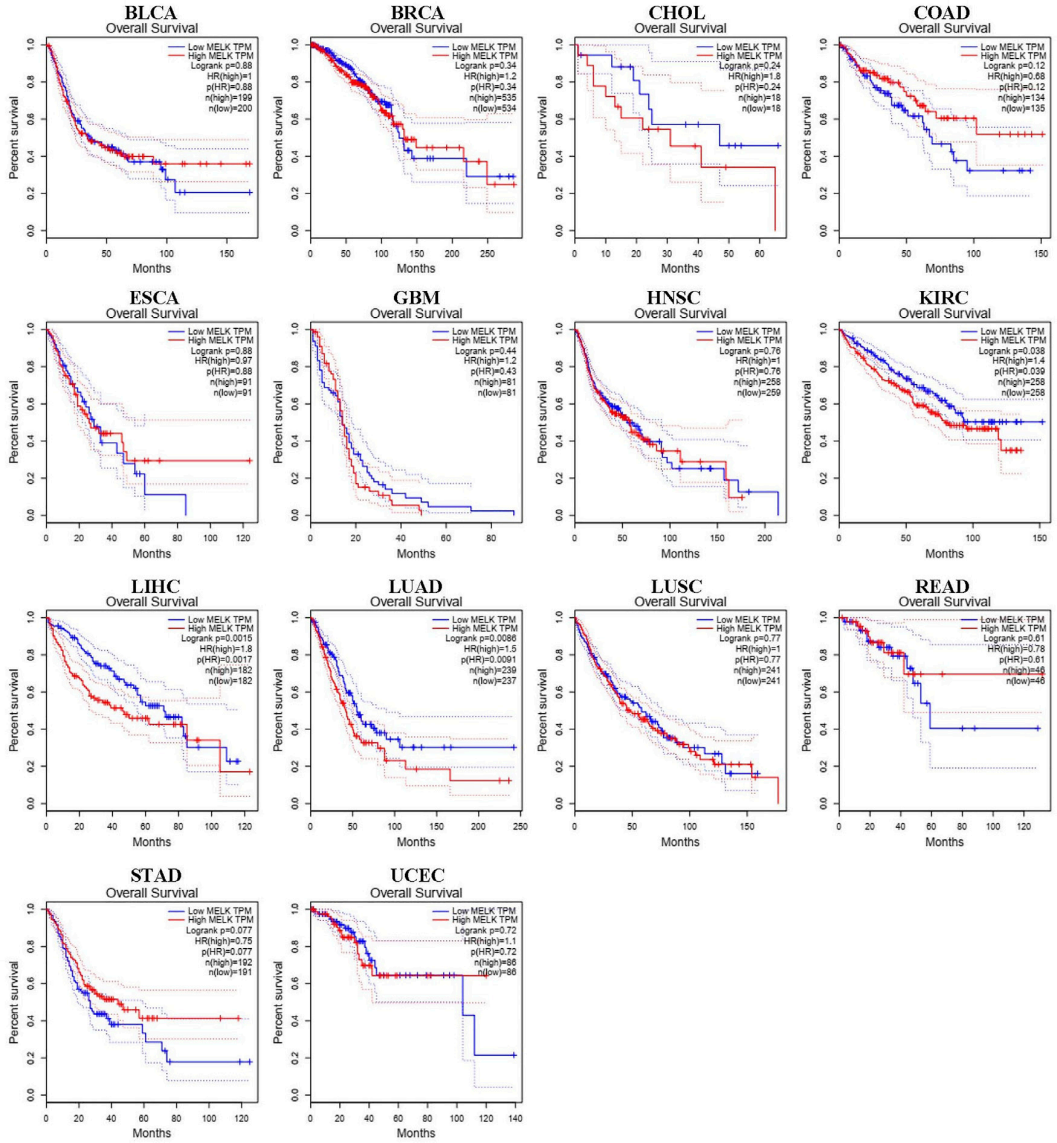
Overall survival (OS) analysis of MELK in various human cancers determined by GEPIA database.

**FIGURE 3:**
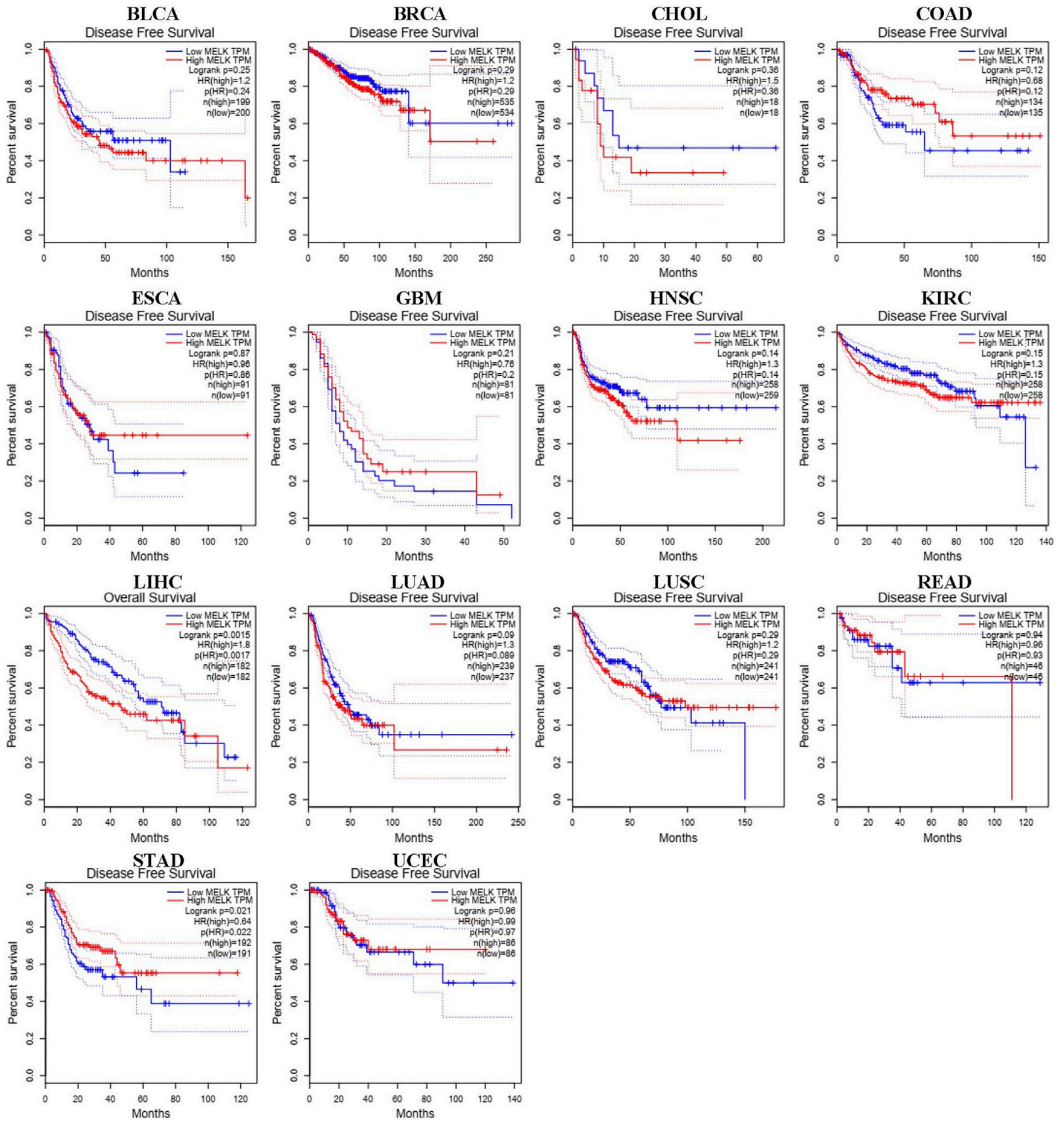
Disease-free survival (DFS) analysis of MELK in various human cancers determined by the GEPIA database.

We used different methods to investigate the prognostic effect of MELK in the 14 types of cancer. The Cox analysis showed that higher expression of MELK was significantly correlated with OS of HCC (HR = 1.79, 95%CI: 1.26∼2.53, *p* = 0.001), LUAD (HR = 1.41, 95%CI: 1.06∼1.89, *p* = 0.02), and KIRC (HR = 1.43, 95%CI: 1.05∼1.94, *p* = 0.022). Then, KM analysis based on the log-rank test was accordingly employed indicating that the higher expression of MELK in LIHC, LUAD, and KIRC predicted a poor prognosis (Figure 2). Then, the DFS was further analyzed and higher expression of MELK indicated poor prognosis in HCC (Figure 3), while no statistical significance of MELK for predicting prognosis of patients in other cancer types was observed. Therefore, MELK may be utilized as a prognostic biomarker in patients with HCC based on the OS and DFS results.

### MELK-Related ceRNA Network Construction in Hepatocellular Carcinoma

**FIGURE 4:**
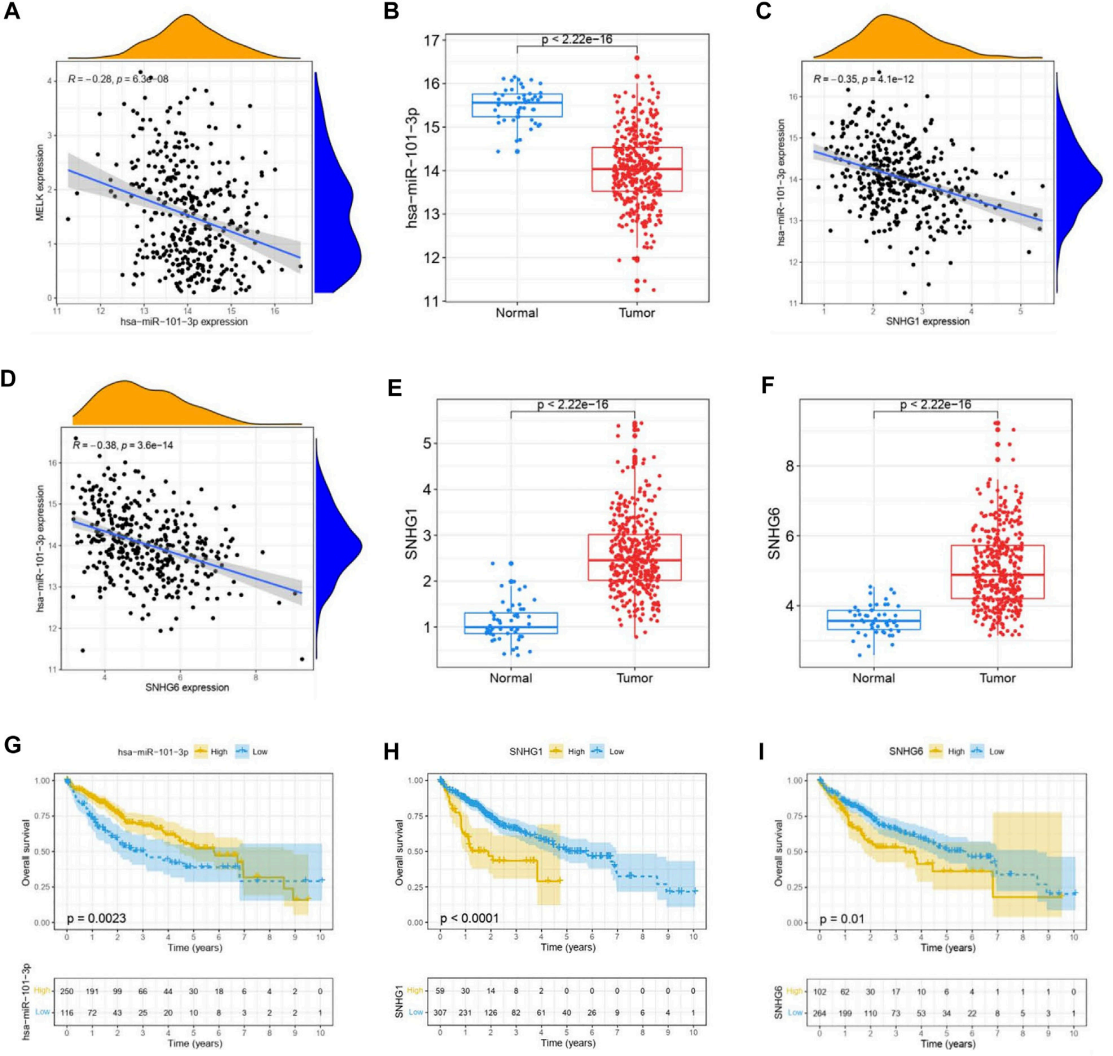
Construction of MELK-associated ceRNA networks. **(A)** Correlation analysis of MELK and hsa-miR-101-3p. **(B)** Expression of hsa-miR-101-3p was compared between HCC and normal samples. **(C)** Correlation analysis between MELK and lncRNAs, SNHG1, and SNHG6. **D)** Differential expression of SNHG1 **(E)** and SNHG6 **(F)** in HCC and normal samples. The survival analysis of hsa-miR-101-3p **(G)**, SNHG1 **(H)**, and SNHG6 **(I)**.

The ceRNA hypothesis suggests that some RNAs possessing miRNA-binding sites can competitively bind to target miRNAs as molecular sponges and subsequently alter the expression of genes encoding proteins (Salmena et al., 2011). It has been widely acknowledged that lncRNAs are responsible for the regulation of gene expression. To ascertain whether MELK was regulated by some lncRNAs, the miRNAs that could potentially bind to MELK were predicted by starBase, and we finally identified 35 miRNAs (Supplementary Table S1). Based on the principle of negative correlation between miRNA and its regulating target gene expression, the correlation between the 35 screened miRNAs and MELK was analyzed. As shown in the Supplementary Tables S1, S7, miRNAs were negatively correlated with MELK in HCC but only hsa-miR-101-3p met the criteria of r < −0.2 and FDR < 0.05 (Figure 4A). Then, the differential expression of hsa-miR-101-3p between HCC samples and normal samples was confirmed (Figure 4B). Taken together, the hsa-miR-101-3p was identified as the most potential regulatory miRNA of MELK in HCC. Also, the binding site between MELK and miR-101-3p is chr9:36677537-36677542 [+].

Next, we used starBase to further predict the lncRNAs that may bind to the hsa-miR-101-3p, and a total of 25 lncRNAs were forecasted. According to the ceRNA hypothesis, lncRNA could upregulate mRNA expression by competitively binding to shared miRNA. Therefore, there should be negative correlation between lncRNA and miRNA or positive correlation between lncRNA and mRNA. As shown in Supplementary Table S2, correlation analysis proved that there are six lncRNAs significantly negatively correlating with hsa-miR-101-3p, namely, SNHG1(Figure 4C), LINC00265, MIR3142HG, GSEC, SNHG6 (Figure 4D), and SNHG14. Of them, SNHG1 (Figure 4E) and SNHG6 (Figure 4F) were differentially expressed between the HCC samples and the normal samples with a log_2_FC > 1 and *p* < 0.05 threshold. The binding site between SNHG1 and miR-101-3p is chr11:62622753-62622772 [-], and the binding site of SNHG6 targeting miR-101-3p is chr8:67834696-67834716 [-]. Then, the correlation of the two lncRNAs and MELK expression was also conducted. As shown in FigureS1A, they both positively correlated with the MELK.

Finally, combining the lncRNA–miRNA pairs and miRNA–mRNA pairs, a lncRNA–miRNA–mRNA network was constructed, including MELK, hsa-miR-101-3p, and two lncRNAs, SNHG1 and SNHG6. The survival analysis of these RNAs grouping by the calculated optimal cutoff was conducted by the KM curve. The results showed that hsa-miR-101-3p (Figure 4G), SNHG1 (Figure 4H), and SNHG6 (Figure 4I) were significantly correlated with OS in HCC. Taking expression analysis and correlation analysis together, SNHG1 and SNHG6 might be the two most potential upstream lncRNAs of the hsa-miR-101-3p/MELK axis in HCC.

### Relationship of MELK Expression and Clinicopathological Features of Hepatocellular Carcinoma Patients

**FIGURE 5:**
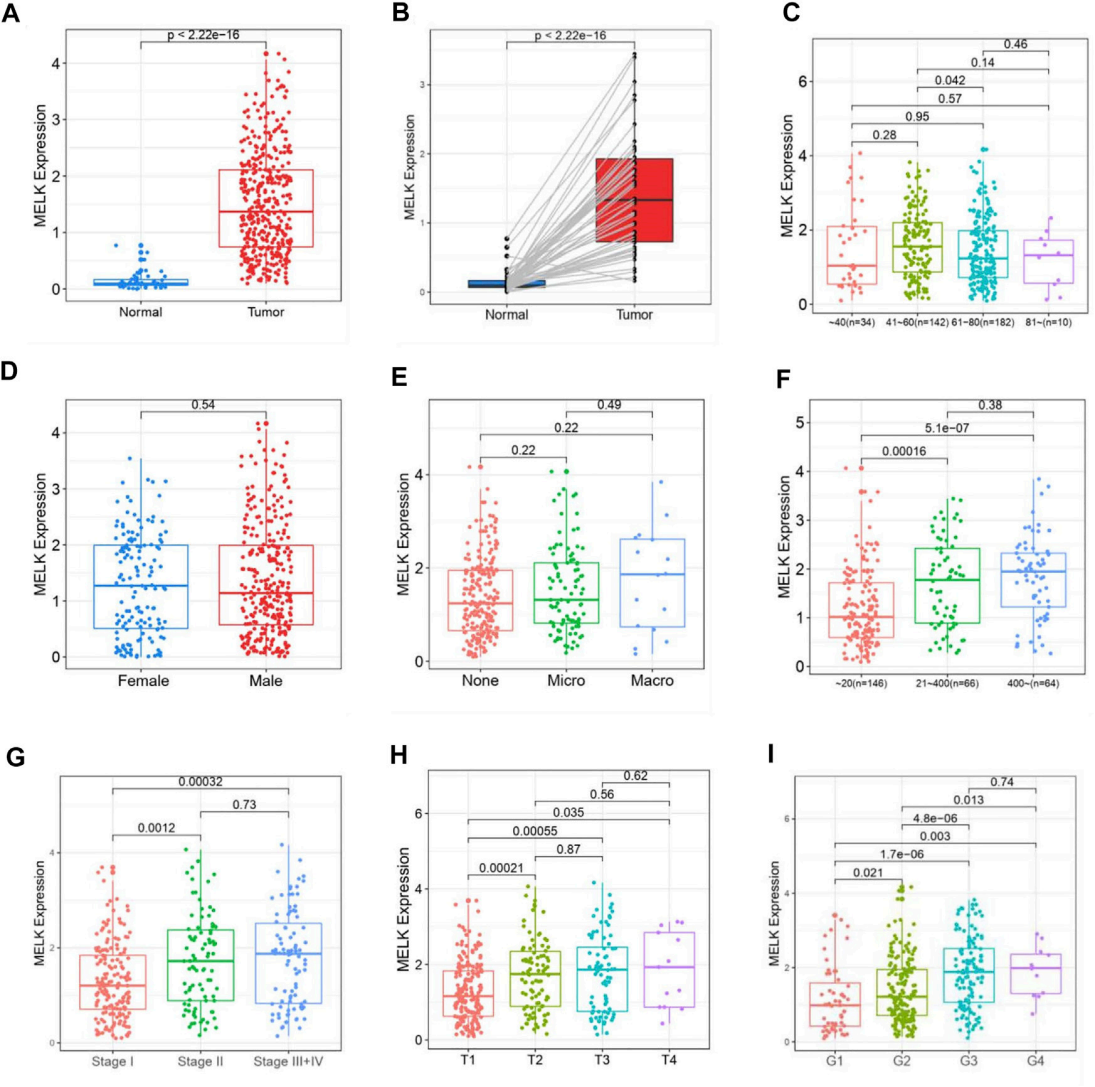
Association of MELK expression and clinicopathological features in HCC. The expression of MELK in HCC was upregulated in both unmatched **(A)** and paired **(B)** normal samples in HCC. Relationship of MELK with age, gender, vascular invasion, clinical stage, T stage, and histological grade **(C–I)**.

The clinicopathological features of HCC patients were obtained from TCGA-LIHC database. Then, we investigated the relationship between the expression of MELK and the clinical features. The expression of MELK in the HCC samples was significantly higher than that in both unmatched (Figure 5A) and paired (Figure 5B) normal samples based on TCGA database. The MELK expression was positively correlated with the AFP level (Figure 5F). The higher level of MELK tended to be correlated with advanced HCC. The Kruskal–Wallis tests revealed that the MELK expression was higher in the advanced clinical stage (Figure 5G), topography (T) stage (Figure 5H), and histological grade (Figure 5I). The univariate and multivariate Cox regression analyses showed that MELK was an independent risk factor for poor prognosis in HCC (Table1). However, the MELK expression was not associated with age, gender, and vascular invasion (Figures 5C–E). The chi-square test was also performed with other clinical information (Supplementary Table S3).

**TABLE 1:** Univariate and multivariate Cox regression analyses of OS/DFS and clinicopathological features in HCC patients.

Overall survival	Univariate analysis		Multivariate analysis	
	HR (95%CI)	*p*-value	HR (95%CI)	*p*-value
Age (≥60 vs. < 60)	1.27 (0.9–1.8)	0.181		
Gender (male vs. female)	1.21 (0.85–1.73)	0.282		
MELK (high vs. low)	1.66 (1.17–2.37)	0.005	1.54 (1.05–2.24)	0.026*
T stage (T3–T4 vs. T1–T2)	2.5 (1.76–3.56)	<0.001	2.3 (1.58–3.35)	<0.001*
Pathologic stage (III–IV vs. I–II)	2.41 (1.66–3.49)	<0.001	1.21 (0.17–8.82)	0.849
Histologic grade (III–IV vs. I–II)	1.14 (0.8–1.63)	0.47		
Child–Pugh grade (B–C vs. A)	1.62 (0.8–3.28)	0.183		
Vascular invasion (positive vs. negative)	1.32 (0.87–2)	0.188		
Family history (yes vs. no)	1.16 (0.81–1.68)	0.415		

Disease-free survival	Univariate analysis		Multivariate analysis	
	HR (95%CI)	*p*-value	HR (95%CI)	*p*-value
Age (≥60 vs. < 60)	1.04 (0.77–1.4)	0.817		
Gender (male vs. female))	0.97 (0.7–1.33)	0.828		
MELK (high vs. low)	1.53 (1.13–2.08)	0.006	1.51 (1.05–2.16)	0.026*
T stage (T3–T4 vs. T1–T2)	2.15 (1.55–2.97)	<0.001	0.33 (0.08–1.4)	0.131
Pathologic stage (III–IV vs. I–II)	2.18 (1.56–3.05)	<0.001	5.82 (1.4–24.16)	0.015*
Histologic grade (III–IV vs. I–II)	1.19 (0.87–1.62)	0.273		
Child–Pugh grade (B-C vs. A)	1.42 (0.78–2.6)	0.253		
Vascular invasion (positive vs. negative)	1.64 (1.16–2.32)	0.005	1.33 (0.91–1.93)	0.141
Family history (yes vs. no)	1 (0.72–1.39)	0.991		

### Characteristics of MELK Immune Cell Infiltration

**FIGURE 6:**
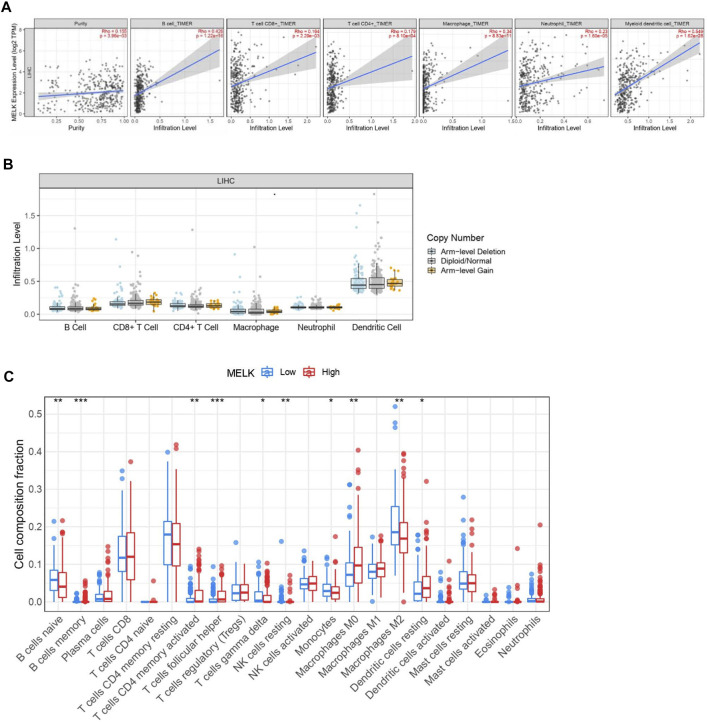
. Relationship of immune cell infiltration with the MELK level in HCC. **(A)** Correlation of the MELK expression level with B cell, CD8^+^ T cell, CD4^+^ T cell, macrophage, neutrophil, and dendritic cell in HCC. **(B)** Infiltration level of various immune cells under different copy numbers of MELK in HCC. **(C)** 22 immune cell subtypes in the high- and low-MELK expression groups in HCC by CIBERSORT. *p < 0.05; **p < 0.01; ***p < 0.001; ****p < 0.0001. ns, not significant.

**FIGURE 7:**
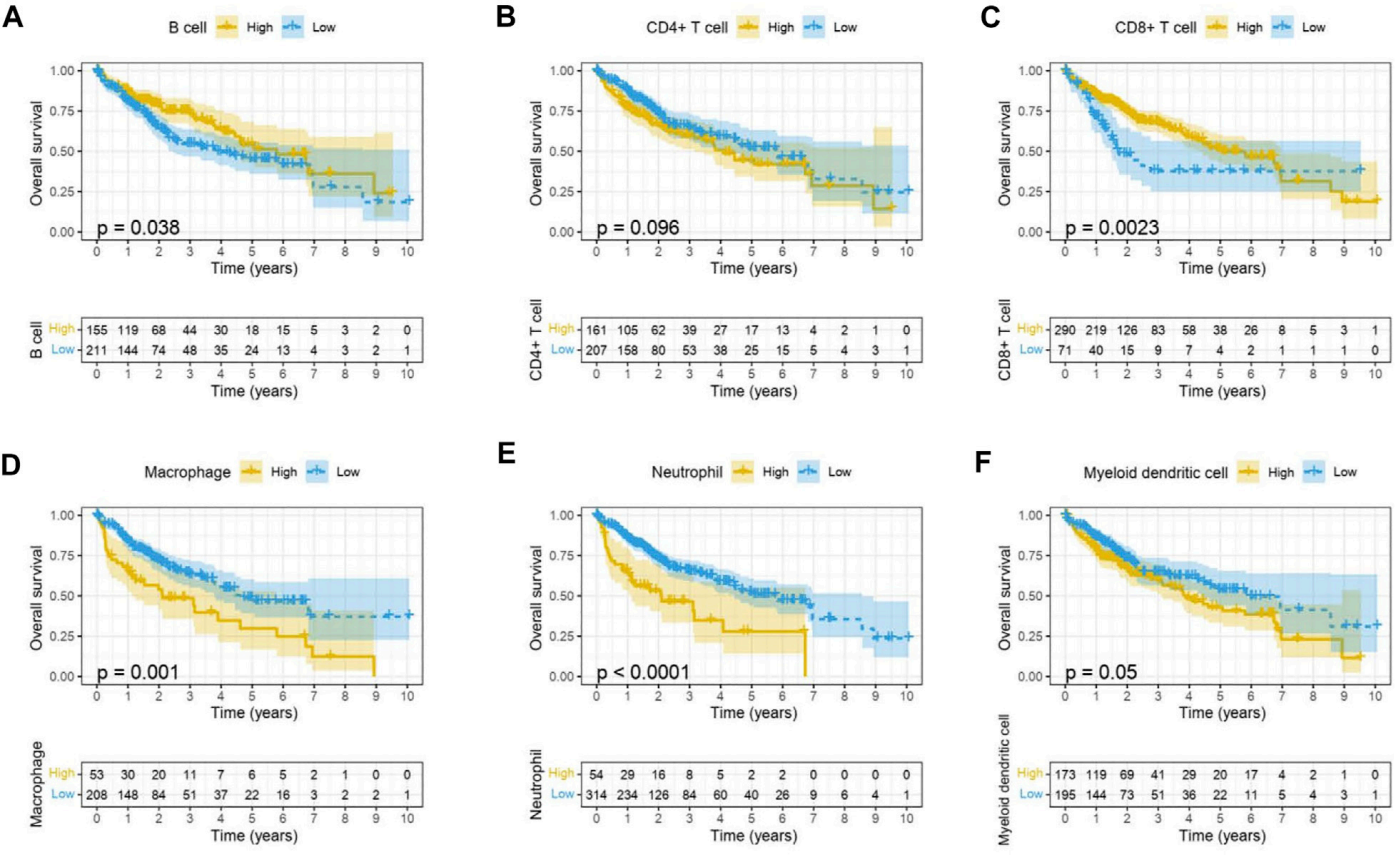
Prognostic value of immune cell infiltration in HCC. B cell **(A)**, CD8^+^ T cell **(B)**, CD4^+^ T cell **(C)**, macrophage **(D)**, neutrophil, **(E)** and dendritic cell **(F)**.

Immune cells in the tumor microenvironment (TME) largely influence the biological behavior of the tumor (Xiong et al., 2018). Previous studies have confirmed that tumor infiltration is associated with the recurrence of HCC (Shi et al., 2011; Gabrielson et al., 2016). Therefore, the correlation of the MELK expression with immune cell infiltration was evaluated using TIMER. As presented in Figure 6A, the MELK expression was significantly positively correlated with all analyzed immune cells, including B cells, CD8^+^ T cells, CD4^+^ T cells, macrophage, neutrophil, and dendritic cell, in HCC. However, the immune cell infiltration level did not alter by various copy numbers of MELK in HCC (Figure 6B). The KM analysis showed that the higher B cells and CD8^+^ T cells were correlated to a better prognosis, while the higher macrophage and neutrophil indicated poor prognosis in HCC (Figures 7A–F). Then, we determined the correlation of MELK and the markers of immune cells using the GEPIA database. The MELK expression was significantly positively correlated with the markers of B cells, M1 macrophage, M2 macrophage, natural killer cell, neutrophil, dendritic cell, and T cells including Treg, Th1, Th2, Tfh, and CD8^+^ T cell (Table2).

**TABLE 2:** Correlation analysis between MELK and biomarkers of immune cells in HCC by the GEPIA database.

Immune cell	Marker	R value	*p*-value
B cell	CD19	0.25	9.8E−07
	CD79A	0.11	3.2E-02
	CD70	0.22	3.0E−05
	CD86	0.3	4.1E−09
CD8^+^ T cell	CD8A	0.17	8.6E-04
	CD8B	0.15	4.0E-03
Treg cells	FOXP3	0.16	1.5E-03
	CCR8	0.38	8.2E−14
	STAT5B	0.33	1.1E−10
	TGFB1	0.21	5.2E−05
Th1	TNF	0.24	4.2E−06
	STAT1	0.38	3.6E−14
	STAT4	0.22	1.4E−05
Th2	IL13	0.12	1.9E-02
	GATA3	0.16	1.5E-03
	STAT5A	0.32	4.9E−10
	STAT6	0.22	2.8E−05
Tfh	CD183	0.22	2.4e−05
	CD185	0.23	5.3e−06
	CD278	0.28	5e−08
Th17	CD360	0.31	1.6e−09
	IL23R	0.18	0.00053
	CD196	0.16	0.0025
M1 macrophage	NOS2	0.032	5.4E-01
	IRF5	0.38	7.4E−14
	PTGS2	0.094	7.2E-02
M2 macrophage	CD163	0.051	3.3E-01
	VSIG4	0.13	1.0E-02
	MS4A4A	0.14	7.4E-03
Neutrophil	CEACAM8	0.12	2.7E-02
	ITGAM	0.35	7.2E−12
	CCR7	0.065	2.1E-01
Dendritic cell	HLA-DPB1	0.18	5.6E-04
	HLA-DRA	0.22	3.0E−05
	HLA-DPA1	0.18	4.7E-04
	CD1C	0.068	1.9E-01
	NRP1	0.26	2.6E−07
	ITGAX	0.33	1.3E−10
Monocyte	CSF1R	0.18	5.9E-04
	CCL2	0.059	2.6E-01
	CD68	0.24	3.4E−06
Natural killer cells	KIR3DL3	0.15	4.9E-03
	KIR2DS4	0.072	1.7E-01
	XCL1	0.31	9.1E−10
	XCL2	0.13	1.3E-02

In addition, 22 types of immune-infiltrating cells were calculated by CIBERSORT. After removing samples that might be computationally inaccurate, the HCC samples were divided into two groups according to the median of MELK expression, the high group and the low group. As shown in the Figure 6C, the naïve B cell, the gamma delta T cell, the resting NK cell, the monocytes, and the M2 macrophage were significantly higher in the low-expression group of MELK, while the memory B cell, the activated CD4 + T cell, the follicular T helper cell, the M0 macrophage, and the resting DC cell were higher in the high-expression group of MELK. The OS analysis of the differential expression of immune cells is shown in Supplementary Figure S2. Altogether, it suggested that MELK may participate in immune response in the tumor microenvironment through affecting immune cells.

### Relationship Between MELK and Immune Checkpoints in Hepatocellular Carcinoma

**FIGURE 8:**
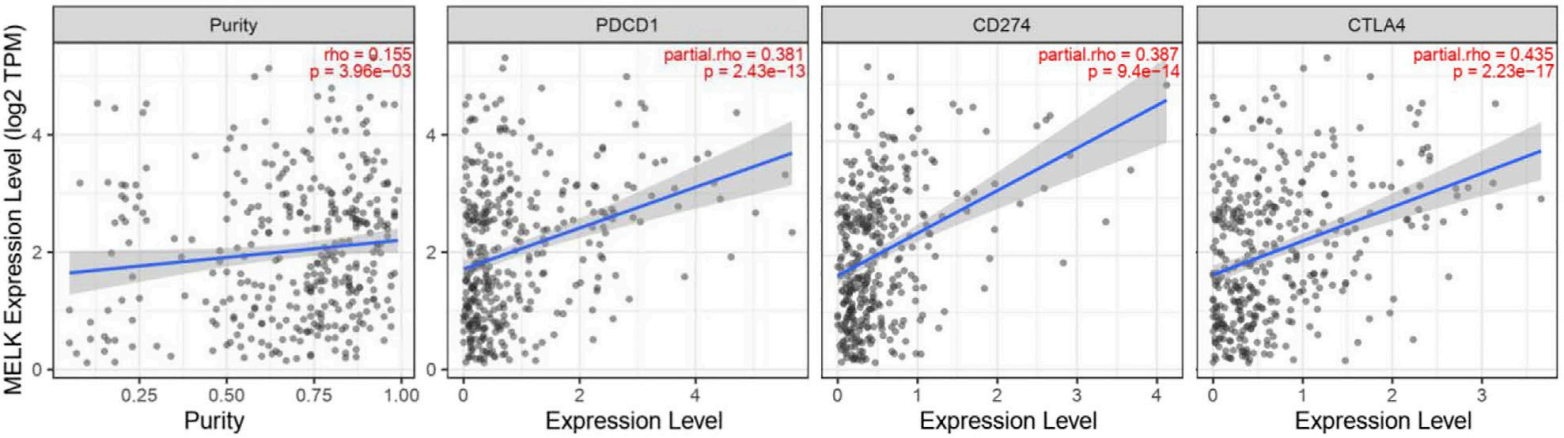
Correlation between MELK and immune checkpoint genes, PD1, PD-L1, and CTLA4.

﻿Immune checkpoints are inhibitory immunoreceptors expressed by effector immune cells that prevent them from becoming overactivated (Liu et al., 2021). ICIs increase anti-tumor immunity by blocking intrinsic downregulators of immunity, such as cytotoxic T lymphocyte antigen 4 (CTLA-4), programmed cell death protein-1 (PD-1), and its ligand programmed cell death-ligand 1 (PD-L1) (Postow et al., 2018). In our study, the correlation between the MELK expression and the immune checkpoint genes was conducted by TIMER. MELK was significantly positively correlated with PD1, PD-L1, and CTLA-4 in HCC (Figure 8). These results provide further evidence that MELK is associated with immunotherapy prognosis.

## Discussion

HCC is one of the most common malignant tumors with high mortality worldwide. The main treatments for HCC include radiofrequency ablation, surgical resection, immunotherapy, and liver implantation. Due to the lack of early clinical symptoms, most HCC patients are generally diagnosed at an advanced stage and lose the timing of better or thorough treatments. They are also at high risk of recurrence and metastasis. Therefore, it is crucial to find effective biomarkers for early diagnosis and prognostic prediction.

It has been reported that MELK is highly upregulated in various types of human cancer according to TCGA dataset and that elevated MELK expression is correlated with poor prognosis of cancer patients (Inoue et al., 2016; Xia et al., 2016). Silencing MELK inhibited the cell growth, invasion, stemness, and tumorigenicity of HCC cells by inducing apoptosis and mitosis (Xia et al., 2016). An orally available MELK inhibitor, OTSSP167, could suppress the growth of GBM (Zhang et al., 2021). This indicated that, especially inhibiting the MELK could be a potential treatment for not only GBM but also other tumors. Nevertheless, the underlying molecular mechanism of MELK affecting HCC is not defined and needs to be further investigated.

We first conducted pan-cancer analysis of the expression of MELK by analyzing RNA-seq data from TCGA database and GEPIA database. MELK was highly expressed in 14 types of tumors including the BLCA, BRCA, CHOL, COAD, ESCA, GBM, HNSC, KIRC, LIHC, LUAD, LUSC, READ, STAD, and UCEC. This suggested that MELK may have a potential promoting role in tumor development. We observed that MELK overexpression significantly indicated the advancement of clinical stages, histological grades, and T stages in HCC patients, and MELK was positively correlated with AFP, the specific HCC marker. For OS and RFS, increased expression of MELK indicated poor prognosis in HCC among all cancer types. Based on the expression analysis and survival analysis, MELK may be utilized as an unfavorable prognostic biomarker for HCC patients.

The ncRNAs, including miRNAs, lncRNAs, and circular RNAs (circRNAs), participated in regulation of gene expression by communicating with each other through the ceRNA mechanism. In this study, we combined the analyses of multiple databases to construct a ceRNA network consisting of lncRNA–miRNA–MELK. A total of thirty five miRNAs that could potentially bind to MELK were predicted by the starBase database. According to our strict screening criteria, hsa-miR-101-3p was chosen as the upstream miRNA. Numerous studies have revealed that miR-101-3p emerges as an essential modulator in the development of human cancers (Ramírez-Salazar et al., 2014; Floor et al., 2015). miR-101-3p has been shown to be downregulated in HCC tissues and cells and could be a potential diagnostic marker of HCC (Yang et al., 2018). In addition, miR-101-3p is responsible for the sensitivity of HCC cells to oxaliplatin by inhibiting Beclin-1-mediated autophagy (Sun et al., 2019). In our study, we found that miR-101-3p was downregulated in HCC patients, and the survival analysis showed a poor prognosis of HCC. Then, we further predicted the upstream lncRNAs of miR-101-3p. A total of twenty five lncRNAs were forecasted. By combining the expression analysis and correlation analysis, two potential upregulated lncRNAs, SNHG1 and SNHG6, were identified. The two lncRNAs have been reported to function as oncogenes in multiple malignancies, including HCC. SNHG1 is upregulated in HCC (Gao et al., 2018), and the knockdown contributed to suppression of HCC cell viability, invasion, and migration properties and promotion of apoptosis, exerting anti-tumor activity in HCC (Meng et al., 2021). SNHG6 is reported to be remarkably increased in HCC tissues and functioned in the proliferation, migration, and invasion abilities of HCC (Fan et al., 2021). The results of the two lncRNAs in our study were consistent with the previous reports. We also found that the higher expression of SNHG1 and SNHG6 was related to poor prognosis and indicated advanced HCC progression (Supplementary Figure S3). Synthetically considering, we suggested that the SNHG1 or SNHG6/miR-101-3p/MELK axis functioned in the HCC progression and could be a potential treatment target in HCC.

In recent decades, convincing evidence has shown that the TME plays an important part in the development of many malignant tumors (Lu J. et al., 2019). The TME of HCC is a complex mixture of hepatic non-parenchymal resident cells, tumor cells, immune cells, and tumor-associated fibroblasts (Sangro et al., 2021). Many types of immune cells are abundant in HCC tumors cooperating in the generation of an immunosuppressive TME, and their presence generally correlates with a worse prognosis (Sangro et al., 2021). Intratumor infiltration of B cells is significantly impaired during HCC progression. The densities of B-cell subsets significantly decreased in the tumor, and high densities of tumor-infiltrating B cells imply a better clinical outcome (Zhang et al., 2019). MELK was significantly positively correlated with the B cells and the markers of B cells. Also, the lower density of B cells predicted a poor prognosis in HCC. Tumor-infiltrating T lymphocytes, especially CD4^+^ and CD8^+^ T cells, play an adaptive immune role in HCC progression. CD8^+^ T cells could recognize tumor-associated antigens and kill tumor cells. The presence of tumor-infiltrating CD8^+^ T cells was significantly correlated with longer OS (Itoh et al., 2020). CD4^+^ T cells could differentiate into Th1, Th2, Th17, Treg cells, and Tfh cells. Treg cells increase in HCC and promote tumor evasion by impairing the function of CD8^+^ T cells and lead to disease progression (Fu et al., 2007). In our study, MELK was positively correlated with the CD8^+^ T cells, CD4^+^ T cells, and the markers of these cells including the subsets of CD4^+^ T cells, Th1, Th2, Th17, Tfh cells, and Tregs. The accumulation of CD8^+^ T cells showed a better prognosis in HCC patients. In the TME, some immune cells could help tumor cells to proliferate, invade, and metastasize. The immunosuppressive features are not only participating in the tumor progression but also a big challenge for effective immunotherapy. The immunosuppressive cells mainly include tumor-associated macrophages (TAMs), tumor-associated neutrophils (TANs), DCs, and Treg cells (Lu C. et al., 2019). MELK was positively correlated with TAMs, TANs, and DCs and the markers of these immune cells. Also, the abundance of TAMs, TANs, and DCs in tumor showed a poor clinical outcome in the HCC patients. This indicated that MELK may involve in the immunosuppression with these immunosuppressive cells.

However, in the CIBERSORT analysis, decreased M2 macrophages were detected in HCC samples (Supplementary Figure S1C) and higher MELK group. Also, better prognosis was found in the group with lower M2 macrophages. Traditionally, macrophages are classified into a classical M1 pro-inflammatory phenotype and a dichotomic M2 anti-inflammatory phenotype (Tacke and Zimmermann, 2014). TAMs are mostly polarized into the M2 phenotype and promote HCC growth, angiogenesis, invasion, and metastasis (Qian and Pollard, 2010). In our work, the analysis of macrophage subsets showed a complicated result remaining to be investigated in further research studies. In addition, CIBERSORT is a convenient and widely used method to determine the composition of immune-infiltrating cells; it is still inaccurate compared with immunohistochemistry and flow cytometry. Overall, the MELK overexpression was significantly correlated with various immune cells, which might indicate its contributions to the immune regulation leading to HCC development.

With the improving understanding of the TME of HCC, the immunotherapy that enhanced tumor immune response and blocked tumor immunosuppression has become a new direction for the treatment of HCC. ICIs targeting PD1, PD-L2, or CTLA-4 are most widely used in the field of advanced HCC (El-Khoueiry et al., 2017; Zhu et al., 2018). PD-1 could transmit inhibitory signals to T cells after binding with PD-L1 or PD-L2 resulting in an immunosuppression and immune tolerance environment and induce the immune escape of tumor cells (Okazaki and Honjo, 2007). CTLA-4 is mainly expressed in activated T cells and dendritic cells and inhibits the activation of T cells, leading to immune escape of HCC cells (Liu and Zheng, 2020). ICIs can cause immune-related adverse events, such as pneumonitis, myocarditis, hypophysitis, and diabetes. It is an important reason preventing the extensive application of ICIs although many patients benefit from the therapy. Therefore, it is of great importance to monitor the treatment efficacy and balance the benefits and side effects for patients receiving ICI therapy. In our study, MELK expression is significantly positively correlated with the immune checkpoint expression, CD274, CTLA4, and PDCD1. This indicated that MELK could predict the efficacy of immunotherapies and provided a direction for new immunotherapy methods.

## Conclusion

In summary, we constructed the MELK–ceRNA network by various bioinformatic analyses which was related to HCC and impacted the prognosis. MELK may also play a vital role in the microenvironment of HCC by regulating various tumor-infiltrating immune cells. In addition, the MELK expression was positively correlated with the immune checkpoint expression. MELK may be a suitable biomarker for HCC patients receiving immunotherapy and can be used as a monitor method to evaluate benefits of patients undergoing ICIs.

## Data Availability

The datasets presented in this study can be found in online repositories. The names of the repository/repositories and accession number(s) can be found in the article/Supplementary Material.
