# Supplementary figures and images for: Comprehensive Analyses of MELK-Associated ceRNA Networks Reveal a Potential Biomarker for Predicting Poor Prognosis and Immunotherapy Efficacy in Hepatocellular Carcinoma
Source: Front Cell Dev Biol. 2022 May 27;10:824938. doi: 10.3389/fcell.2022.824938 (PMC9184526; doi:10.3389/fcell.2022.824938)

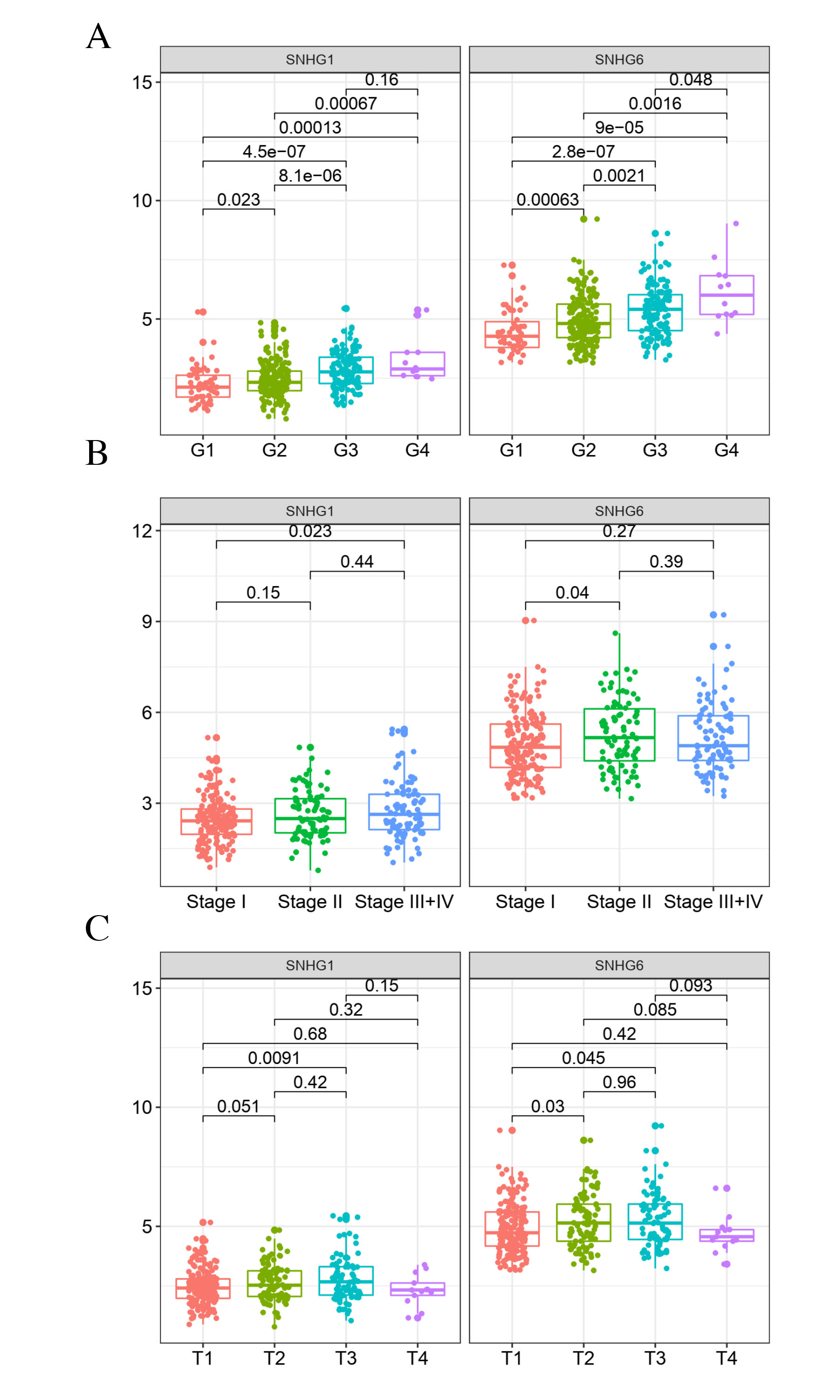

Supplement: Supplementary file 1 [file Image3.JPEG]

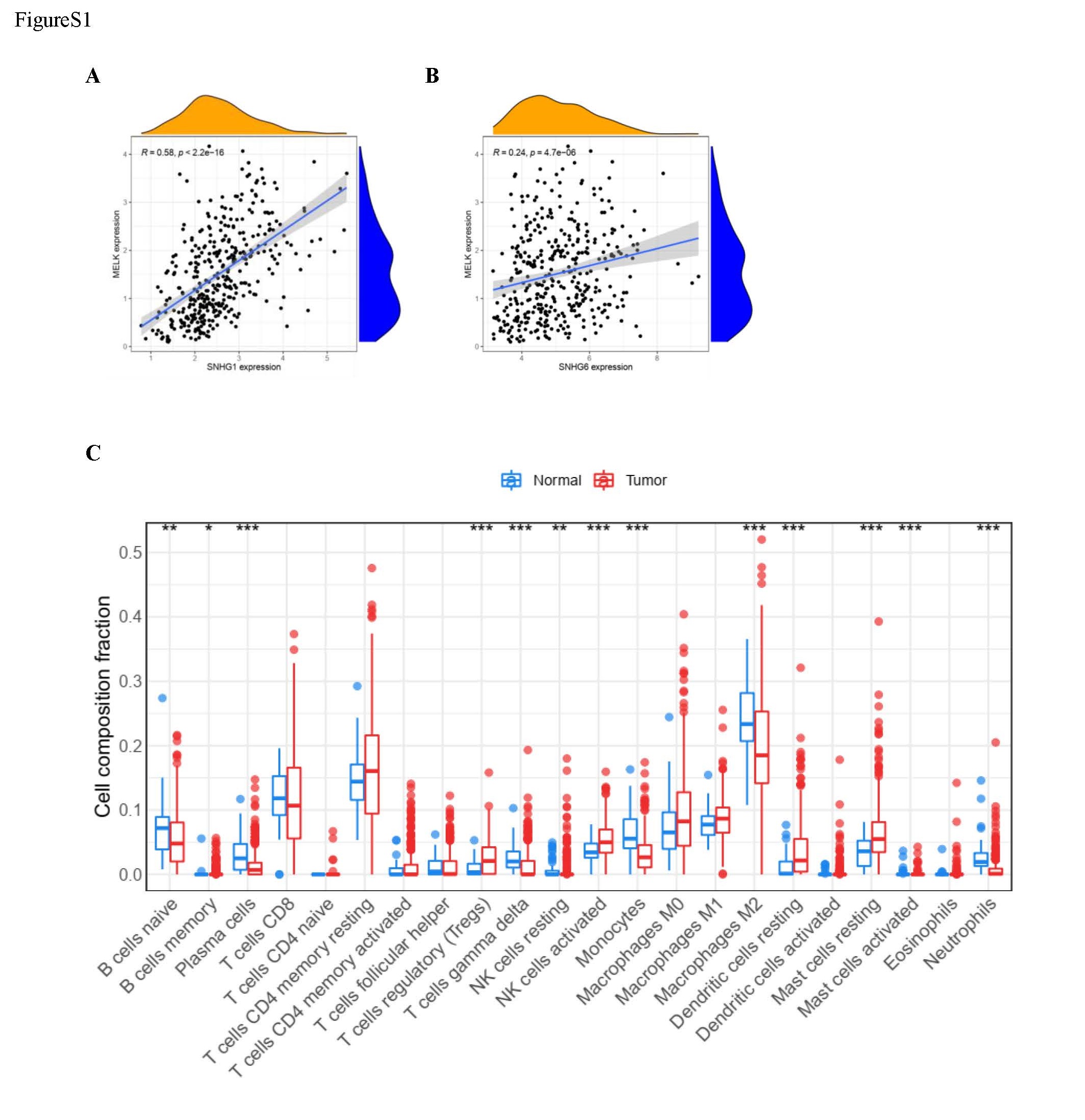

Supplement: Supplementary file 3 [file Image1.JPEG]

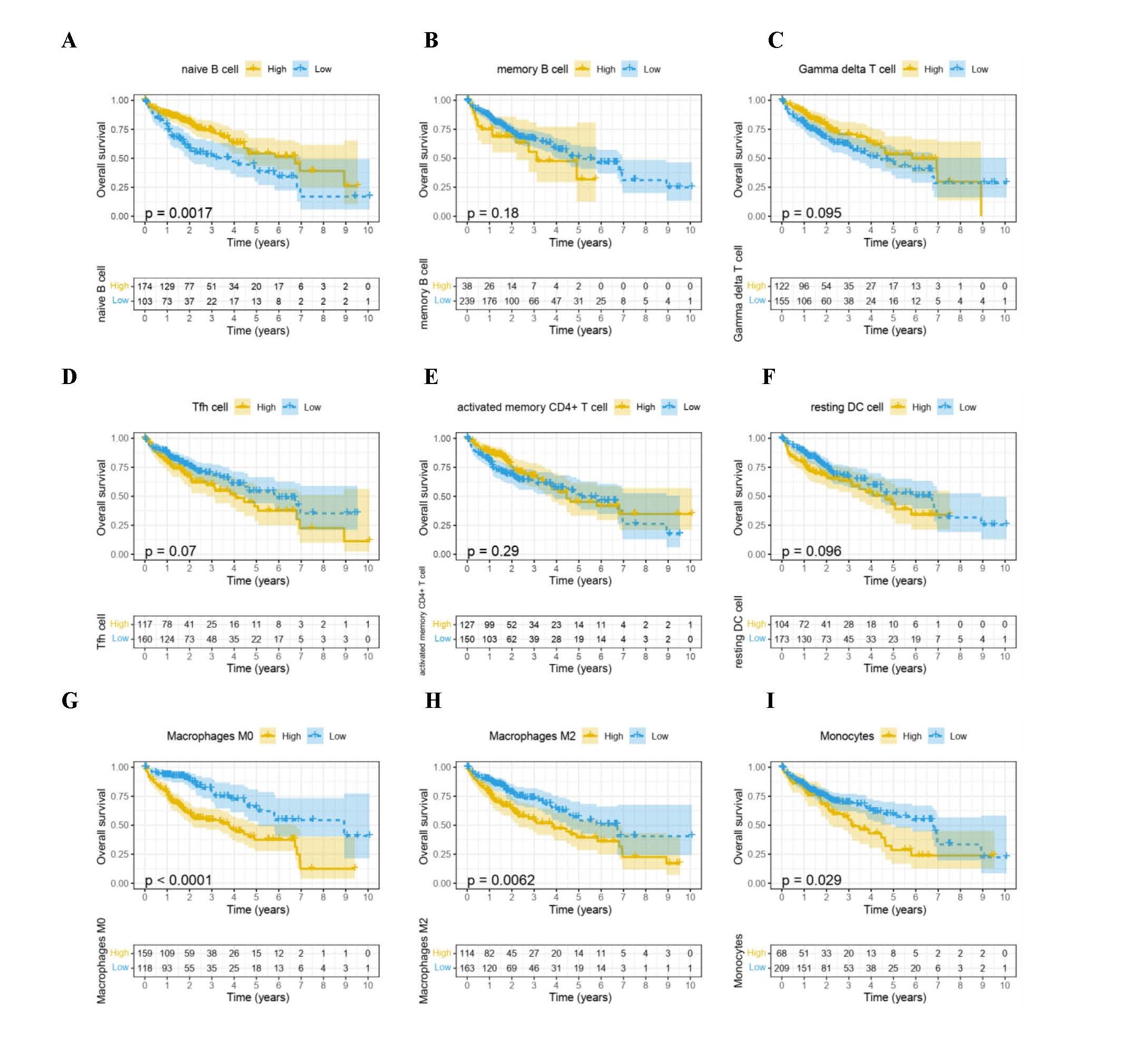

Supplement: Supplementary file 4 [file Image2.JPEG]
