# Supplementary material for: Comprehensive Analyses of MELK-Associated ceRNA Networks Reveal a Potential Biomarker for Predicting Poor Prognosis and Immunotherapy Efficacy in Hepatocellular Carcinoma
Source: Front Cell Dev Biol. 2022 May 27;10:824938. doi: 10.3389/fcell.2022.824938 (PMC9184526; doi:10.3389/fcell.2022.824938)
Supplement: Supplementary file 2 [file Table1.DOCX]

Table S1. The correlation analysis between MELK and miRNA and differential expression of the miRNAs.

| Gene | miRNA | R value | pvalue | logFC | diffPval |
| --- | --- | --- | --- | --- | --- |
| **MELK** | **hsa-miR-101-3p** | **-0.277877259** | **6.25E-08** | **-1.472341348** | **1.33E-24** |
| MELK | hsa-miR-194-5p | -0.182856645 | 0.000415866 | -0.280569014 | 0.158127645 |
| MELK | hsa-miR-144-3p | -0.091759934 | 0.077943136 | -2.642568758 | 1.05E-22 |
| MELK | hsa-miR-133a-3p | -0.077950426 | 0.134494525 | -0.409317189 | 0.001174245 |
| MELK | hsa-miR-224-3p | -0.071625056 | 0.169181047 | 0.97774941 | 5.58E-08 |
| MELK | hsa-miR-23b-3p | -0.057564434 | 0.269263399 | -0.514071046 | 5.62E-09 |
| MELK | hsa-miR-505-3p | -0.005401615 | 0.917487104 | -1.167274939 | 3.39E-15 |
| MELK | hsa-miR-133b | 0.001172363 | 0.982069408 | -0.492755131 | 4.26E-13 |
| MELK | hsa-miR-485-3p | 0.050322652 | 0.334388573 | -0.192017767 | 0.001884985 |
| MELK | hsa-miR-224-5p | 0.052048038 | 0.317903068 | 2.573873219 | 4.10E-16 |
| MELK | hsa-miR-382-5p | 0.067077465 | 0.197877342 | -0.550939205 | 4.79E-06 |
| MELK | hsa-miR-944 | 0.075826477 | 0.145472071 | 0.212007668 | 0.944255751 |
| MELK | hsa-miR-376c-3p | 0.077917507 | 0.134659652 | -1.372027611 | 2.08E-14 |
| MELK | hsa-miR-513b-5p | 0.08167733 | 0.11678775 | 0.020061285 | 0.473432375 |
| MELK | hsa-miR-181c-5p | 0.089825109 | 0.084444156 | 0.037095837 | 0.719955643 |
| MELK | hsa-miR-181d-5p | 0.102596805 | 0.048641274 | 0.194058913 | 0.149291266 |
| MELK | hsa-miR-381-3p | 0.119926606 | 0.021084041 | -1.05412489 | 6.74E-11 |
| MELK | hsa-miR-889-3p | 0.120080122 | 0.020918901 | -0.608533236 | 7.54E-08 |
| MELK | hsa-miR-23a-3p | 0.127521868 | 0.01414977 | -0.162025574 | 0.146204125 |
| MELK | hsa-miR-181a-5p | 0.141203772 | 0.0065171 | 0.271489616 | 0.00503133 |
| MELK | hsa-miR-802 | 0.159291045 | 0.002117036 | -0.230523527 | 4.50E-08 |
| MELK | hsa-miR-323a-3p | 0.163694865 | 0.001580851 | -0.790561588 | 5.77E-11 |
| MELK | hsa-miR-181b-5p | 0.189432967 | 0.000253686 | 0.500459568 | 2.27E-05 |
| MELK | hsa-miR-524-5p | 0.203864162 | 7.83E-05 | 0.298845813 | 0.014515743 |
| MELK | hsa-miR-21-5p | 0.205155349 | 7.28E-05 | 1.509435455 | 5.22E-23 |
| MELK | hsa-miR-548o-3p | 0.21548959 | 2.91E-05 | 0.249705937 | 0.000162951 |
| MELK | hsa-miR-520d-5p | 0.221871987 | 1.65E-05 | 0.193787139 | 0.076251221 |
| MELK | hsa-miR-186-5p | 0.239215338 | 3.49E-06 | -0.041756245 | 0.355073706 |
| MELK | hsa-miR-1276 | 0.242483032 | 2.37E-06 | 0.253363894 | 9.50E-06 |
| MELK | hsa-miR-212-3p | 0.276992425 | 6.08E-08 | -0.16208823 | 0.134981095 |
| MELK | hsa-miR-132-3p | 0.279655015 | 5.12E-08 | 0.341680651 | 0.000182956 |
| MELK | hsa-miR-522-3p | 0.302810877 | 2.76E-09 | 0.407662769 | 0.039765275 |
| MELK | hsa-miR-590-5p | 0.342100341 | 1.81E-11 | -0.086403981 | 0.119119235 |
| MELK | hsa-miR-421 | 0.346460947 | 7.12E-12 | 0.665797793 | 3.33E-09 |
| MELK | hsa-miR-425-5p | 0.350682324 | 5.19E-12 | 0.601201316 | 6.79E-07 |

Table S2. The correlation analysis between hsa-miR-101-3p and the predicted lncRNAs and differential expression of the lncRNAs.

| lncRNA | miRNA | R value | pvalue | logFC | diffPval |
| --- | --- | --- | --- | --- | --- |
| **SNHG6** | **hsa-miR-101-3p** | **-0.381255267** | **3.63E-14** | **1.451472559** | **3.16E-21** |
| GSEC | hsa-miR-101-3p | -0.379632213 | 4.88E-14 | 0.374516828 | 2.66E-18 |
| **SNHG1** | **hsa-miR-101-3p** | **-0.352288558** | **4.09E-12** | **1.457690034** | **4.47E-26** |
| SNHG14 | hsa-miR-101-3p | -0.268967159 | 1.67E-07 | 0.296678112 | 0.006128952 |
| LINC00265 | hsa-miR-101-3p | -0.267875619 | 1.68E-07 | 0.16591967 | 1.79E-06 |
| FAM201A | hsa-miR-101-3p | -0.262699058 | 2.95E-07 | 0.15855717 | 0.706224475 |
| MIR3142HG | hsa-miR-101-3p | -0.252726728 | 8.44E-07 | 0.137124453 | 8.36E-05 |
| PTPRG-AS1 | hsa-miR-101-3p | -0.164277893 | 0.001520067 | 0.067521462 | 6.40E-09 |
| RUSC1-AS1 | hsa-miR-101-3p | -0.147892854 | 0.004393291 | 0.911796314 | 1.14E-24 |
| LINC00997 | hsa-miR-101-3p | -0.13381011 | 0.010017168 | 0.414373865 | 8.01E-16 |
| LINC00943 | hsa-miR-101-3p | -0.131331898 | 0.011450769 | 0.025985214 | 1.45E-05 |
| SND1-IT1 | hsa-miR-101-3p | -0.127569664 | 0.014065384 | 0.002474362 | 0.312079421 |
| MALAT1 | hsa-miR-101-3p | -0.122563863 | 0.018399105 | 1.047743425 | 1.04E-15 |
| SNHG21 | hsa-miR-101-3p | -0.087679672 | 0.092156942 | 0.412063735 | 6.92E-21 |
| LINC00891 | hsa-miR-101-3p | -0.06955133 | 0.181897034 | -0.001731968 | 0.067125415 |
| XIST | hsa-miR-101-3p | -0.068270944 | 0.190093153 | 0.012765208 | 0.675672464 |
| LINC01579 | hsa-miR-101-3p | -0.052244077 | 0.316237777 | 0.019326148 | 0.002219083 |
| DSCAM-AS1 | hsa-miR-101-3p | -0.036783635 | 0.480566317 | -0.006258662 | 0.324670475 |
| PSMA3-AS1 | hsa-miR-101-3p | -0.034506204 | 0.507993341 | 0.566011143 | 5.76E-14 |
| NEAT1 | hsa-miR-101-3p | -0.030717576 | 0.555689066 | 0.929263755 | 9.87E-09 |
| EBLN3P | hsa-miR-101-3p | 0.03738179 | 0.473276042 | 0.388929474 | 3.52E-06 |
| TYMSOS | hsa-miR-101-3p | 0.061497577 | 0.237861241 | 0.923230503 | 3.63E-14 |
| RN7SL832P | hsa-miR-101-3p | 0.1377774 | 0.007956701 | 0.102450256 | 0.000292515 |
| SNHG22 | hsa-miR-101-3p | 0.180227917 | 0.000504397 | 0.209511184 | 1.02E-07 |
| GABPB1-AS1 | hsa-miR-101-3p | 0.258011361 | 5.34E-07 | 0.246797891 | 1.62E-11 |

TableS3. Correlation of MELK expression with clinicopathological features in the TCGA cohort

| **Characteristic** | **Low (186)** | **High (185)** | **Pvalue** |
| --- | --- | --- | --- |
| **Age, n (%)** |  |  | 0.062 |
| <=60 | 80 (21.6%) | 97(26.3%) |  |
| >60 | 106(28.6%) | 87 (23.5%) |  |
| **Gender, n (%)** |  |  | **0.055** |
| Female | 52 (14.1%) | 69 (18.6%) |  |
| Male | 134 (36.1%) | 116 (31.2%) |  |
| **Pathologic stage, n (%)** |  |  | **< 0.001** |
| Stage I | 102 (29.4%) | 69 (19.9%) |  |
| Stage II | 36 (10.4%) | 50 (14.4%) |  |
| Stage III | 30 (8.6%) | 55 (15.9%) |  |
| Stage IV | 4 (1.2%) | 1 (0.2%) |  |
| **T stage, n (%)** |  |  | **< 0.001** |
| T1 | 109(29.6%) | 72 (19.6%) |  |
| T2 | 38 (10.3%) | 56 (15.2%) |  |
| T3 | 30 (8.2%) | 50 (13.6%) |  |
| T4 | 6 (1.6%) | 7 (1.9%) |  |
| **N stage, n (%)** |  |  | 0.624 |
| N0 | 120 (46.9%) | 132 (51.6%) |  |
| N1 | 1 (0.4%) | 3 (1.2%) |  |
| **M stage, n (%)** |  |  | 0.622 |
| M0 | 129 (47.8%) | 137 (50.7%) |  |
| M1 | 3 (1.1%) | 1 (0.4%) |  |
| **Histologic Gade, n (%)** |  |  | **< 0.001** |
| G1 | 39(10.7%) | 16(4.4%) |  |
| G2 | 101(27.6%) | 76(20.8%) |  |
| G3 | 40(10.9%) | 82(22.4%) |  |
| G4 | 4(1.1%) | 8(21.9%) |  |
| **AFP (ng/ml), n** **(%)** |  |  | **< 0.001** |
| <=400 | 126 (45.3%) | 87 (31.4%) |  |
| >400 | 19 (6.8%) | 46 (16.5%) |  |
| **Child-Pugh grade, n (%)** |  |  | 0.702 |
| A | 119 (49.8%) | 98 (41.0%) |  |
| B | 12 (5.0%) | 9 (3.8%) |  |
| C | 1 (0.4%) | 0 (0%) |  |
| **Fibrosis ishak score, n (%)** |  |  | 0.279 |
| 0 | 45 (21.2%) | 29 (13.7%) |  |
| 1/2 | 14 (6.6%) | 17 (8%) |  |
| 3/4 | 12 (5.7%) | 16 (7.5%) |  |
| 5/6 | 44 (20.8%) | 35(16.5%) |  |
| **Vascular invasion, n (%)** |  |  | 0.463 |
| No | 111 (35.2%) | 95 (30.2%) |  |
| Yes | 54 (17.1%) | 55 (17.5%) |  |
