# Supplementary material for: Comprehensive Analyses of MELK-Associated ceRNA Networks Reveal a Potential Biomarker for Predicting Poor Prognosis and Immunotherapy Efficacy in Hepatocellular Carcinoma
Source: Front Cell Dev Biol. 2022 May 27;10:824938. doi: 10.3389/fcell.2022.824938 (PMC9184526; doi:10.3389/fcell.2022.824938)
Supplement: Supplementary file 5 [file DataSheet1.PDF]

Supplementary Material

FigureS1

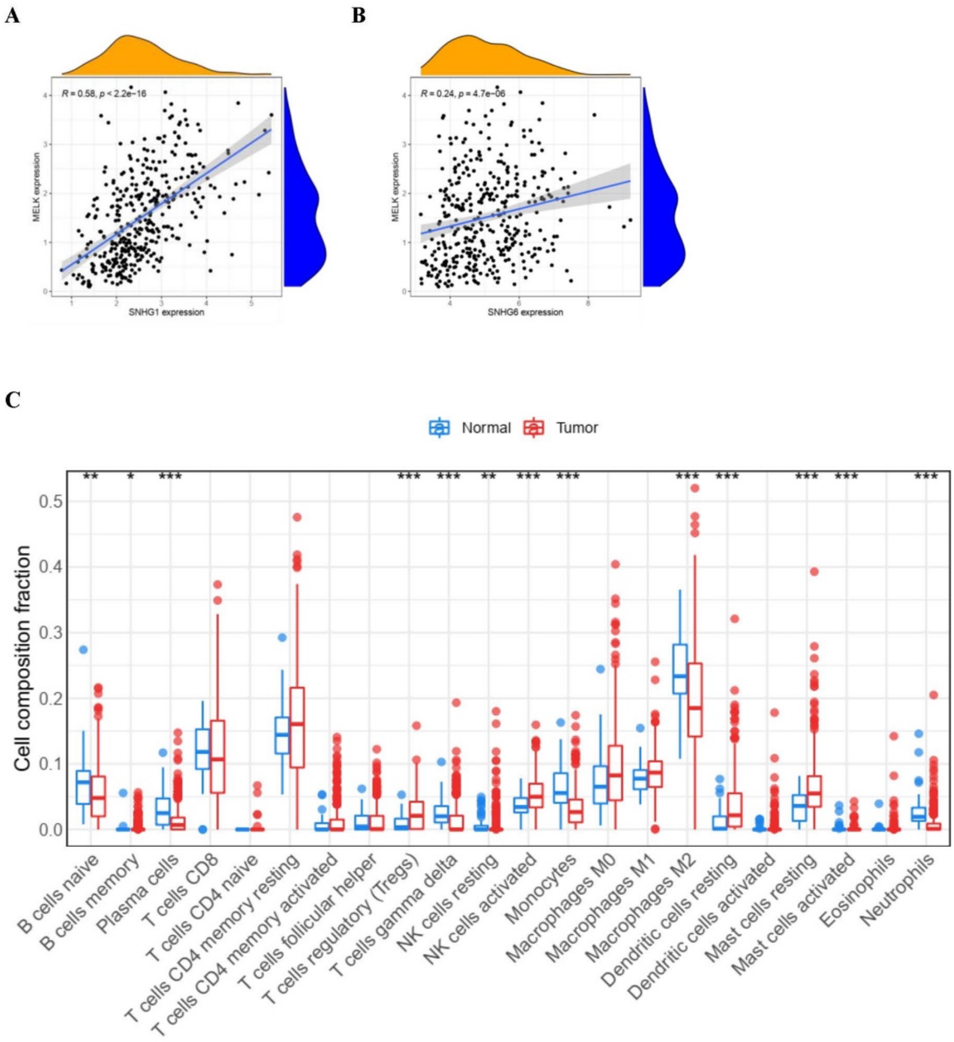

Supplementary Figure 1. The correlation of MELK and lncRNA, SNHG1(A) and SNHG6(B). (C)The 22 immune cell subtypes in HCC and normal samples by CIBERSORT. *p < 0.05; **p < 0.01; ***p < 0.001; ****p <0.0001. ns, not significant.

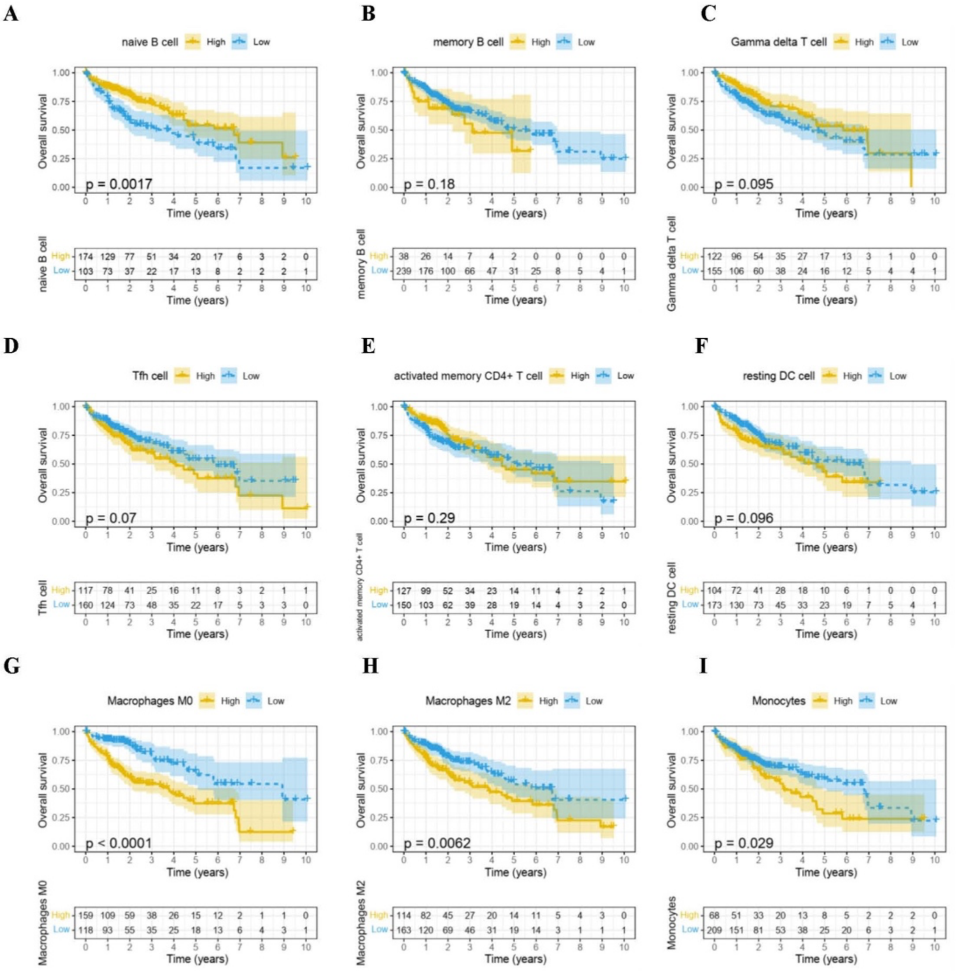

Supplementary Figure 2. The prognostic value of immune cell infiltration in HCC.

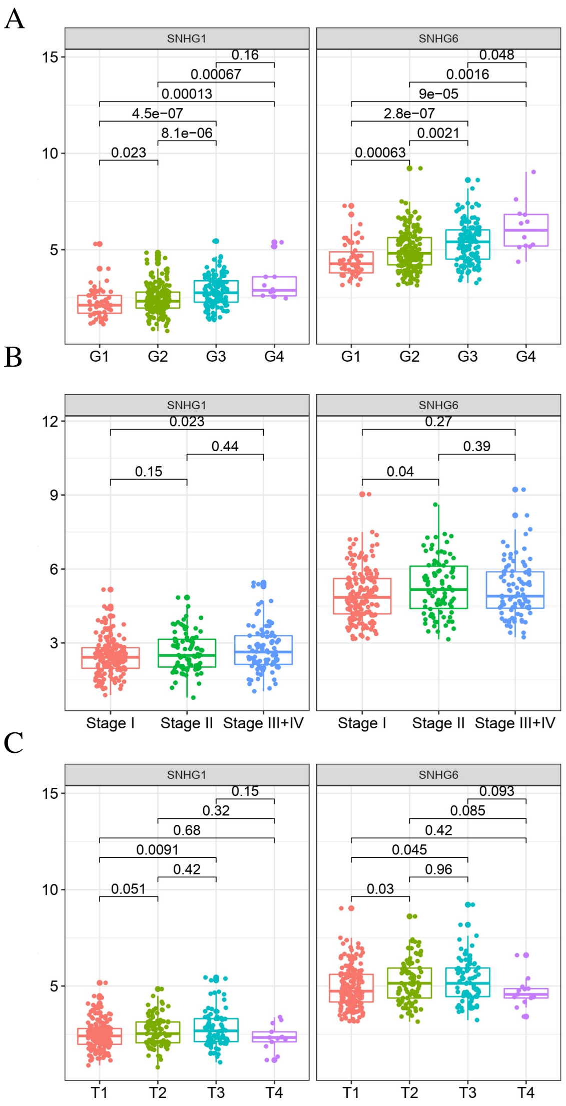

Supplementary Figure 3. The correlation between the lncRNA expression and histological grade(A), clinical stage(B)and T stage (C).
